# Suicidal behaviour and intravenous drug use in chemsex context

**DOI:** 10.1192/j.eurpsy.2024.615

**Published:** 2024-08-27

**Authors:** J. Curto Ramos, A. Rodríguez Laguna, P. Barrio, L. Ibarguchi, A. García, I. Azqueta, H. Dolengevich Segal

**Affiliations:** ^1^Department of Psychiatry, Clinical Psychology and Mental Health, La Paz University Hospital; ^2^ Apoyo Positivo; ^3^Dual Disorders Program. Department of Psychiatry, Henares University Hospital, Madrid, Spain

## Abstract

**Introduction:**

Several studies have called atention to the mental health disorders associated with chemsex --the intentional use of drugs before or during sexual intercourse GBMSM (gay, bisexual and men who have sex with men) population-. Sexualized intravenous drug use is also known as slam or slamsex. There are few studies that analyze the mental health differences between intravenous drug users compared to non-intravenous drug users in chemsex context.

**Objectives:**

To describe the suicidal behaviour in a sample of users with sexualized drug use (chemsex) attended by the non-governmental organization Apoyo Positivo in the program “Sex, Drugs and You” and to compare the suicidal behaviour between intravenous drug users compared to non-intravenous drug users.

**Methods:**

A cross-sectional descriptive analysis of a sample of users attended by the non-governmental organization Apoyo Positivo in the program “Sex, Drugs and You” between 2016-2019 was performed.

**Results:**

We included 217 participants. 37 had attempted suicide at least once. The percentage of chemsex users who have attempted suicide were significantly higher in the intravenous drug use group compared to the non-intravenous drug use group (p<0.05).

**Conclusions:**

Possible risk factors for suicidal behaviour among chemsex users include slamsex. Other possible risk factors previously described in other studies include adversities experienced due to one’s sexual orientation and an increased risk for HIV and other STI infections. Further studies analyzing the relationship between chemsex, slamsex and suicidal behaviour are needed.

**Disclosure of Interest:**

None Declared

